# ITScan: a web-based analysis tool for Internal Transcribed Spacer (ITS) sequences

**DOI:** 10.1186/1756-0500-7-857

**Published:** 2014-11-27

**Authors:** Milene Ferro, Erik A Antonio, Wélliton Souza, Maurício Bacci

**Affiliations:** Centro de Estudos de Insetos Sociais, Instituto de Biociências, UNESP - Univ Estadual Paulista, Rio Claro, SP 13506-900 Brazil; Departamento de Bioquímica e Microbiologia, Instituto de Biociências, UNESP - Univ Estadual Paulista, Rio Claro, SP 13506-900 Brazil; Departamento de Ciência da Computação, Universidade Federal de São Carlos, São Carlos, SP 13565-905 Brazil

**Keywords:** Fungal biodiversity, Mycology, Pipeline, Web service

## Abstract

**Background:**

Studies on fungal diversity and ecology aim to identify fungi and to investigate their interactions with each other and with the environment. DNA sequence-based tools are essential for these studies because they can speed up the identification process and access greater fungal diversity than traditional methods. The nucleotide sequence encoding for the internal transcribed spacer (ITS) of the nuclear ribosomal RNA has recently been proposed as a standard marker for molecular identification of fungi and evaluation of fungal diversity. However, the analysis of large sets of ITS sequences involves many programs and steps, which makes this task intensive and laborious.

**Findings:**

We developed the web-based pipeline ITScan, which automates the analysis of fungal ITS sequences generated either by Sanger or Next Generation Sequencing (NGS) platforms. Validation was performed using datasets containing ca. 2,000 to 40,000 sequences each.

**Conclusions:**

ITScan is an online and user-friendly automated pipeline for fungal diversity analysis and identification based on ITS sequences. It speeds up a process which would otherwise be repetitive and time-consuming for users. The ITScan tool and documentation are available at http://evol.rc.unesp.br:8083/itscan.

## Findings

### Background

Studies on fungal biodiversity use DNA sequence-based tools to generate molecular marker to identify rare species and determine associations in a microbial community[1]. The technique is particularly powerful in characterizing fungal diversity in environmental samples containing many fungal species which do not grow, or grow poorly, in laboratory cultures[2]. Many biodiversity studies are based on the nuclear ribosomal Internal Transcribed Spacer (ITS) region[3, 4], which is a small (~500 base-pair) region occurring in multiple copies in the fungal nuclear genome and shows a high degree of variation even between closely related species[5].

The ITS region has been recently designated as a universal marker for molecular barcoding of fungi[1] or the default region for species identification. To determine the microbial diversity in environmental samples, generated ITS sequences are grouped in operational taxonomic units (OTUs), often using the MOTHUR program[6] and an OTU-based approach analysis[7, 8]. The use of multiple programs and stages of analysis make the process laborious and time-consuming. In this work, we describe a web-based pipeline that automates the study of fungal diversity and identification based on ITS sequences.

## Implementation

### Architecture design

**Figure 1:**
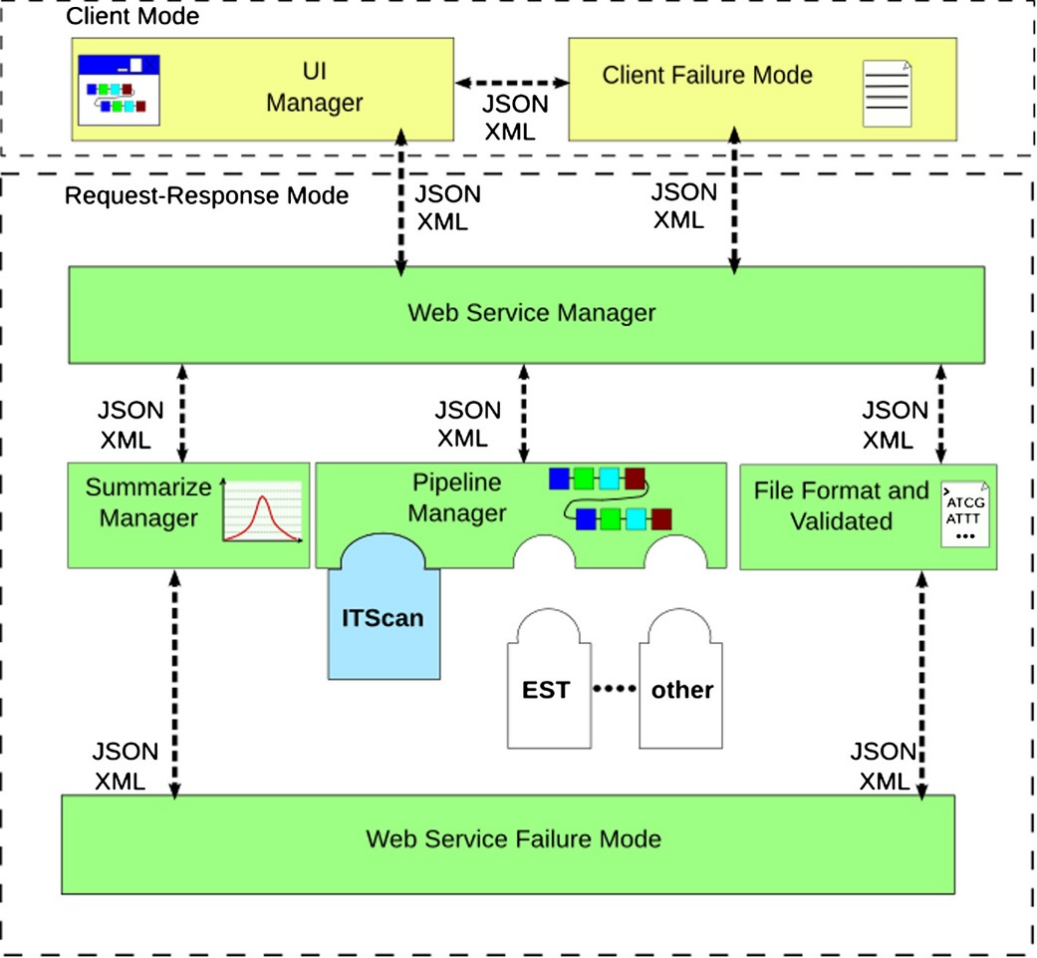
**System architecture that coupled ITScan.** The figure displays the ITScan architecture model based on MVC (Model-View-Controller) and J2EE design patterns. The architecture model was tailored to represent two main viewpoints: Client Mode and Request-Response Mode.

We developed an architectural model based on MVC (Model-View-Controller) and J2EE design patterns[9] (Figure 1). The architectural model also depicts two base formats for data interchange: JavaScript Object Notation (JSON) and Extensible Markup Language (XML). These formats represent data and functions as well as each step used in the pipeline architecture to perform fungal analysis. The architecture model was tailored to represent two main viewpoints:

- Client Mode — aims at dealing with client-side concerns;
- Request-Response Mode — performs a set of server-side and business logic concerns using coupled third-party programs and their business rules. The Pipeline Manager provides Representation State Transfer - REST[10] service.

This architecture assists background information to check for failures in client and server sides.

### Pipeline for fungal ITS analysis

ITScan requires a FASTA-formatted input file containing pre-processed sequences, i.e., high quality sequences (usually Phred ≥20) without primer and adaptor sequences. Pre-processing programs, such as SEQTRIM[11], SCATA[12], PANGEA[13], CANGS[14] and PYRONOISE[15], can be used to trim data from different sequencing platforms (e.g. 454, Illumina, regular Sanger reads) and the resulting output files can then be read by ITScan.

**Figure 2:**
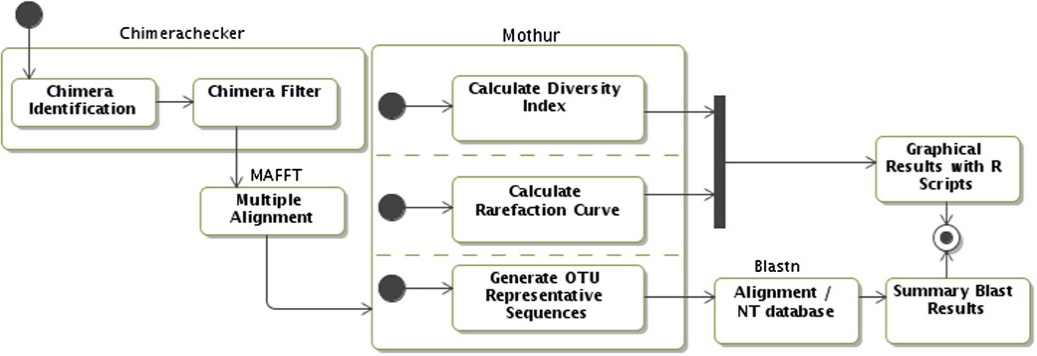
**State machine diagram describing ITScan pipeline steps.** The third-party programs were integrated in the pipeline as shown by the state machine diagram using UML. Each program in ITScan is a web service developed using REST.

The third-party programs ChimeraChecker[16], MAFFT[17], MOTHUR and BLAST[18] were integrated in the pipeline as shown by the state machine diagram using UML[19] (Figure 2). Each program in ITScan is a web service developed using REST technology, which was shown to improve client usability[20, 21]. In the first step, ChimeraChecker is used to classify all sequences as chimeric, non-chimeric or not evaluated using default parameters. Non-chimeric ITS sequences are then aligned to each other in the MAFFT software. Aligned sequences are run into the MOTHUR package, which clusters similar sequences to each other to generate operational taxonomic units (OTUs), and calculates diversity indexes and richness estimators[6]. User can set the ITScan label parameter to define the dissimilarity value (%) that represents the maximal percentage of difference between the sequences in the same OTU. MOTHUR selects a representative sequence which has the smallest distance from all remaining sequences within a given OTU. The selected representative sequence (or centroid) is used in a BLASTN search and the first hit is used to identify the OTU. The utilization of a centroid instead of all sequences composing the OTU speeds up computation processing. BLAST results are presented in tabular format with links to GenBank.

## Results

The architectural model enables the user to develop web service components and to couple them in a new customized pipeline. R language scripts provide graphic results and spreadsheets representing rarefaction curves as well as Shannon or Simpson diversity indexes and Chao1 richness estimator.

ITScan has a user-friendly interface and can process up to three a FASTA-formatted input files simultaneously and compare these files with each other. The pipeline was validated using Sanger sequences (Mantovani et al., in preparation) and a large dataset (2,000 to 40,000 sequences) simulating results from Next Generation Sequencing (NGS), which was retrieved from the UNITE[22] database.

Many programs which analyze ITS fungal sequences, such as FungalITSPipeline[23], QIIME[24] and FHiTINGS[25], require the user installation and operation via command line. These requirements are not necessary in ITScan, which was built with a web-based interface.

The ITScan pipeline comes with some limitations. For instance, it processes only three FASTA files simultaneously. In addition, it relies on GenBank servers to run BLASTN searches, instead of implementing time-consuming local searches on annotated databases[22] which would improve taxonomic assignment. Future expansions in our servers will allow us to implement multi sample analyses based on local annotated fungal ITS databases.

## Conclusions

This work describes an architectural model that can be used with bioinformatics third-party programs. All components follow the same framework, which facilitates the development of new components. ITScan works with sequences derived from both Sanger and NGS technologies. The pipeline can process single or as many as three datasets to compare distinct biological samples. Output data include graphs and spreadsheets that are automatically generated to represent fungal diversity. ITScan includes an user manual and an example dataset. We validated ITScan using datasets containing ca. 2,000 and 40,000 sequences retrieved from the UNITE database. Using of ITScan does not require computational expertise.

## Availability and requirements

**Project name:** ITScan

**Project home page:**http://evol.rc.unesp.br:8083/itscan

**Operating system(s):** Platform independent

**Programming language:** Perl, Java

**Other requirements:** Web browser

**License:** ITScan web tool is freely available for all users. ITScan is open source under the GNU GPL license.
